# *MSH2* is the very young onset ovarian cancer predisposition gene, not *BRCA1*

**DOI:** 10.1136/jmg-2022-109055

**Published:** 2023-03-09

**Authors:** Nicola Flaum, Emma J Crosbie, Emma Roisin Woodward, Fiona Lalloo, Robert Morgan, Neil Ryan, D Gareth Evans

**Affiliations:** 1 Division of Evolution, Infection and Genomics, School of Biological Sciences, Faculty of Biology, Medicine and Health, University of Manchester, Manchester Academic Health Science Centre, Manchester, UK; 2 North West Genomics Laboratory Hub, Manchester Centre for Genomic Medicine, St Mary’s Hospital, Manchester University NHS Foundation Trust, Manchester Academic Health Science Centre, Manchester, UK; 3 Division of Cancer Sciences, School of Medical Sciences, Faculty of Biology, Medicine and Health, University of Manchester, Manchester, UK; 4 Division of Gynaecology, St Mary’s Hospital, Manchester University NHS Foundation Trust, Manchester, UK; 5 Clinical Genetics Service, Manchester Centre for Genomic Medicine, St Mary’s Hospital, Manchester University NHS Foundation Trust, Manchester, UK; 6 The Christie NHS Foundation Trust, Manchester, UK; 7 The Academic Women’s Health Unit, Translational Health Sciences, Bristol Medical School, University of Bristol, Bristol, UK; 8 Prevention Breast Cancer Centre and Nightingale Breast Screening Centre, University Hospital of South Manchester, Manchester, UK; 9 Manchester Breast Centre, Manchester Cancer Research Centre, University of Manchester, Manchester, UK

**Keywords:** Congenital, Hereditary, and Neonatal Diseases and Abnormalities, Genetic Counselling, Genetic Predisposition to Disease, Genetic Testing, gynaecology

## Referenced paragraph

The genetic cause of very young onset ovarian cancer (VYOC), diagnosed under 30 years of age, is unclear.^1^ The histology and underlying genetics in VYOC is significantly different from the overall epithelial ovarian cancer (EOC) population; we aimed to explore this in VYOC cases known to the North-West of England. We found mismatch repair genes to be the most commonly affected in VYOCs, especially *MSH2*. The cumulative likelihood of an EOC in *MSH2* heterozygotes is >2% by age 35, with this likelihood still below 0.5% for *BRCA1* and rare for *BRCA2*.^2^

## Article

The inherited landscape of epithelial ovarian cancer (EOC) is well established with contributions from homologous recombination deficiency (HRD) genes, particularly *BRCA1* and *BRCA2*, and mismatch repair deficiency (MMRD) genes *MSH2*, *MLH1*, *MSH6* and *PMS2*.^1^ High-grade serous ovarian cancer (HGSOC) is associated with HRD, accounting for up to 23% of HGSOC.^2^ Approximately 3% of EOC cases occur in <30 years of age, described as very young onset ovarian cancer (VYOC).^1^ The pathology in VYOC differs from overall EOC; a study of 114 VYOC cases found only 28% were serous, while 59% had mucinous pathology.^3^ Among the 101 tested cases, no *BRCA1* or *BRCA2* pathogenic variant (PV) was identified, only 2 *MLH1* PVs.^3^ VYOC seems associated with MMRD-related EOC as opposed to homologous recombination deficiency (HRD)-related EOC as is seen in *BRCA1/2* carriers, in whom the risk of EOC increases from 35 years for *BRCA1* carriers and from 45 years in *BRCA2* carriers.^2 4^

We retrospectively assessed the presence of PVs in VYOC cases aged <30 and 30–34 years before the main risk period is associated with PVs in *BRCA1/2*. These women had been referred to Manchester Centre for Genomic Medicine (MCGM) within the last two decades following diagnosis with VYOC in the North-West of England. The genetic testing described was performed as part of standard diagnostic testing within MCGM.^5^ A series of 77 women with ovarian cancer (9 borderline and 1 granulosa cell tumour in addition to EOCs) were screened for MMRD and HRD germline PVs by sequencing, multiple ligation-dependent probe amplification and a prescreen for MMR immunohistochemistry (IHC) as previously described.^5^ We also assessed the proportion of known carriers that developed EOC at age <30 and 30–34 years from our extensive dataset of >4000 female *BRCA1/2* carriers and 910 MMRD heterozygotes.

Of the 77 ovarian cancer cases aged <30 years, no *BRCA1/2* PV was identified. However, five MMRD PVs (four *MSH2*, one *PMS2*) were detected, making up 6.5% of all cases and 7.5% of epithelial cases, as shown in table 1. Of the 69 invasive tumours, 5 were of unspecified histological subtype (1x*MSH2*), 2 clear cell (1x*MSH2*), 7 endometrioid (1x*MSH2*, 1x*PMS2*), 29 mucinous and 12 serous tumours not otherwise specified (1x*MSH2*). Age range was 15.0–29.9; mean age=25.00, median age=25.77, IQR=23.23–28.00. The age of the four *MSH2* heterozygotes was 23.6, 25.1, 26.2 and 27.5 years and that of *PMS2* homozygote was 26.8 years.

**Table 1:** Breakdown of VYOC cases by histological subtype

Histological subtype	n (%)	Carriers of PV detected (%)	PV details
HGSOC	6 (7.8)	0	NA
LGSOC	4 (5.2)	0	NA
Poorly differentiated	1 (1.3)	0	NA
Serous NOS	12 (15.6)	1 (8.3)	MSH2x1
Endometrioid	7 (9.1)	2 (28.6)	MSH2x1, PMS2x1
Clear cell	2 (2.6)	1 (50)	MSH2x1
Mucinous	29 (37.7)	0	NA
Borderline	9 (11.7)	0	NA
Granulosa cell	1 (1.3)	0	NA
Adenocarcinoma NOS	1 (1.3)	0	NA
Unknown	5 (6.5)	1 (20)	MSH2x1
**Total**	**77**	**5** (**6.5%**)	**MSH2x4, PMS2x1**

HGSOC, high-grade serous ovarian cancer; LGSOC, low-grade serous ovarian cancer; NA, Not Applicable; NOS, not otherwise specified; PV, pathogenic variant; VYOC, very young onset ovarian cancer.

When assessing the proportions of VYOC in PV carriers of HRD and MMRD genes, we included all tested and obligate carriers. There were 2005 female *BRCA1,* 1999 *BRCA2* and 393 *MSH2* PV heterozygotes (table 2). One *BRCA1* PV carrier (obligate carrier) was identified with an EOC at age <30 (0.05%) (26 years old at diagnosis) but pathology subtype was unavailable. In contrast to the low rate in *BRCA1/2* carriers, 4 out of 393 VYOC cases (1%) were found to carry the same *MSH2* PV. This proportion was significantly greater in *MSH2* PV carriers than either *BRCA1* (p=0.003) or *BRCA2* (p=0.0007) by χ^2^ testing.

**Table 2:** Proportion of women with VYOC with heterozygous PVs in *BRCA1/2* and MMRD genes

Gene	Female carriers (n)	OC <30 (%)	OR	P value	OC 30–34 (%)	OR	P value
*BRCA1*	2005	1 (0.05)	0.05	0.003	6 (0.30)	0.23	0.02
*BRCA2*	1999	0 (0.00)	NA	0.007	3 (0.15)	0.12	0.004
*MSH2*	393	4 (1.02)	Ref	NA	5 (1.29)	Ref	NA
*MLH1*	278	0 (0.00)	NA	NA	0 (0.00)	NA	0.013
*MSH6*	169	0 (0.00)	NA	NA	0 (0.00)	NA	0.06
*PMS2*	70	1 (1.43)	NA	NA	0 (0.00)	NA	0.36

MMRD, mismatch repair deficiency; NA, Not Applicable; OC, ovarian cancer; PV, pathogenic variant; Ref, reference population; VYOC, very young onset ovarian cancer.

Of the 2005 *BRCA1* and 1999 *BRCA2* carriers, six and three cases of ovarian cancer were diagnosed, respectively, between 30 and 34 years. This was significantly less than the five cases found in 393 *MSH2* PV carriers (p=0.02; 0.004, respectively). The proportion of ovarian cancer in *MSH2* PV carriers <35 years was significantly higher than 0 out of 278 found for *MLH1* PV carriers. In addition to the previous four cases, there were two clear cell, one endometrioid, one serous and one yolk sac tumour (non-EOC, but with MSH2 loss detected by IHC in tumour) histological subtypes. Only one *MSH2* PV carrier died from EOC with 77% surviving 10 years and 66% surviving >15 years.

In contrast, 7 out of 10 *BRCA1/2* PV heterozygotes had died, with 4 died <5 years of diagnosis. Two long-term *BRCA2* survivors both diagnosed at aged 32 had mucinous tumours (the remaining histologies were high-grade serous: *BRCA1*=2, adenocarcinoma not otherwise specified *BRCA1*=5; endometrioid *BRCA2*=1), raising doubts as to whether these were HRD-driven tumours. Hypothesising that these mucinous cases were not HRD-driven, survival at 12 years was significantly better in *MSH2* (77%) than *BRCA1/2* heterozygotes (15%; p=0.01).

Very few studies have addressed the contribution of HRD and MMRD genes to VYOC. In addition to the study of 101 cases aged <30,^3^ we identified a study of 47 women diagnosed at age ≤40 with EOC who underwent germline screening for 11 genes associated with ovarian cancer. This identified PVs in 13 (28%) of women (*BRCA1*: 10, *BRCA2*: 1, *MSH2*: 1, *RAD51D*: 1).^6^ This study included only two women diagnosed under 30 years of age, neither of whom had an identifiable PV.

Our study has shown that while the genetic predisposition for many early onset ovarian cancers is still unknown, *MSH2* is the most important EOC predisposition gene at age <35 years. The cumulative likelihood of an EOC in *MSH2* heterozygotes would appear to be >2% by 35, with this likelihood still below 0.5% for *BRCA1* and rare for *BRCA2* 2; indeed, two-thirds of cases identified in *BRCA2* carriers may not have been driven by HRD. This increased incidence despite the good long-term survival in *MSH2* should prompt awareness of the increased risk and consideration for early risk-reduction strategies.
